# Comparative genomics analysis of the companion mechanisms of *Bacillus thuringiensis* Bc601 and *Bacillus endophyticus* Hbe603 in bacterial consortium

**DOI:** 10.1038/srep28794

**Published:** 2016-06-29

**Authors:** Nan Jia, Ming-Zhu Ding, Feng Gao, Ying-Jin Yuan

**Affiliations:** 1 Key Laboratory of Systems Bioengineering (Ministry of Education), School of Chemical Engineering and Technology, Tianjin University, Tianjin, 300072, PR China; 2 SynBio Research Platform, Collaborative Innovation Centre of Chemical Science and Engineering (Tianjin), School of Chemical Engineering and Technology, Tianjin University, Tianjin, 300072, PR China; 3 Department of Physics, Tianjin University, Tianjin, 300072, PR China

## Abstract

*Bacillus thuringiensis* and *Bacillus endophyticus* both act as the companion bacteria, which cooperate with *Ketogulonigenium vulgare* in vitamin C two-step fermentation. Two *Bacillus* species have different morphologies, swarming motility and 2-keto-L-gulonic acid productivities when they co-culture with *K. vulgare*. Here, we report the complete genome sequencing of *B. thuringiensis* Bc601 and eight plasmids of *B. endophyticus* Hbe603, and carry out the comparative genomics analysis. Consequently, *B. thuringiensis* Bc601, with greater ability of response to the external environment, has been found more two-component system, sporulation coat and peptidoglycan biosynthesis related proteins than *B. endophyticus* Hbe603, and *B. endophyticus* Hbe603, with greater ability of nutrients biosynthesis, has been found more alpha-galactosidase, propanoate, glutathione and inositol phosphate metabolism, and amino acid degradation related proteins than *B. thuringiensis* Bc601. Different ability of swarming motility, response to the external environment and nutrients biosynthesis may reflect different companion mechanisms of two *Bacillus* species. Comparative genomic analysis of *B. endophyticus* and *B. thuringiensis* enables us to further understand the cooperative mechanism with *K. vulgare*, and facilitate the optimization of bacterial consortium.

The microbial communities have been deemed important organisms based on their metabolic capabilities and potential applications^1^. The co-cultured organisms can divide the labor reasonable and improve the robustness of the system^2^. For example, the ability to metabolize and degrade cellulose^3^, alkanes^4^, heavy metal toxins^5^ and natural products^6^. The multiple microbial species increased range of genes and metabolic capabilities in comparison to monocultures. Enumerating metabolic exchanges and the microbial diversity are still uncharacterized for complex metabolic needs and biosynthetic capabilities.

The microbial community of *K. vulgare* and *Bacillus* species have been widely used in the two-step vitamin C production^7^. In the community, *K. vulgare* is responsible for the bioconversion of sorbose to 2-keto-L-gulonic acid (2-KLG, the precursor of vitamin C). Mono-cultured *K. vulgare* grows poorly, even on rich natural media. Addition of amino acids^8^, vitamins^9^ and glutathione^10^ could enhance the growth of *K. vulgare*, implying the defects of oxidation metabolism, vitamin and amino acid synthesis^11^. The *Bacillus* species have been used to stimulate the growth of *K. vulgare*, including *Bacillus megaterium*, *Bacilius cereus* and *B. endophyticus*, and different 2-KLG productivities have been determined in the co-culture systems. Besides, mix cultured *B. megaterium* and *B. cereus* (1:3) could increase the 2-KLG yield compared to one-helper-strain co-culture system^12^. In our previous studies, the metabolomics approach has been demonstrated on the interactions between *K. vulgare* and *Bacillus*^10^,^13^. However, the different cooperative effects and mechanisms of companion bacteria are needed to interpret at molecular level.

Comparative genomics is a powerful approach for studying variation in physiological traits as well as the evolution and ecology of microorganisms^14^. Based on comparative genomics analysis, relationship of *B. megaterium* with other *Bacillus* species and numerous unique genetic traits were identified^15^. Through comparative genomics, the evolutionary relationships and major traits of *Bacillus* species were understood, including the diversity of sporulation and competence genes^16^. Whole-genome sequencing of *B. thuringiensis* 97-27 and *B. cereus* E33L was undertaken to identify shared and unique genes, the differences were revealed in terms of virulence, structural components and regulatory mechanisms^17^.

Identification the optimum companion bacterium has always been an important research topic in VC two-step fermentation. In present study, we observe the different phenotype of *B. thuringiensis* Bc601 and *B. endophyticus* Hbe603 and different 2- KLG productivities of *K. vlugare* in the co-culture system with different helper bacteria. In order to understand the genome evolution and the metabolic versatility, we have sequenced the complete genomes of *B. thuringiensis* Bc601 and eight plasmids of *B. endophyticus* Hbe603. Aided with comparative genomics analysis, characteristics of two species and cooperation mechanisms could be systematically understood, which are of great importance for consortium optimization.

## Results

### General genomic properties of two *Bacillus* species

The complete genome of *B. thuringiensis* Bc601 consists of one circular chromosome and six plasmids (Fig. S1 and Table S1). *B. anthracis*, *B. cereus* and *B. thuringiensis* are closely-related members, which have been named as the *B. cereus*-group. Comparison of their 16S rRNA sequences places them within the same species^18^. *B. thuringiensis* and *B. cereus* mix together in the phylogenetic tree by blasting the whole genome sequences. *B. thuringiensis* Bc601 is most similar with *B. thuringiensis serovar kurstaki* HD73, which contains 15 contigs (Fig. S2). The genes in plasmids are mainly encodes transposases, transcriptional regulators, recombinases, type VII secretion proteins and cell surface proteins. Besides, *B. thuringiensis* Bc601 also encodes thiamine biosynthesis protein, 3-oxoacyl-ACP synthase, ATPase and flagellar biosynthesis protein on plasmids for nutrients synthesis.

The complete genome of *B. endophyticus* Hbe603 consists of one circular chromosome and eight plasmids (Table S2). Our previous research published the chromosome of *B. endophyticus* Hbe603^19^. Besides, it owns eight plasmids, which have the special sequence information and low similarity compared to diverse *Bacillus spp*. With the RAST analysis platform, we identify 468 genes in eight plasmids and almost half of them encode the hypothetical proteins or unknown enzymes. Six plasmids contain phage proteins which may derive from horizontal transfer. The genes in plasmids are mainly about sporulation, germination, regulation and transportation. The sporulation cycle is complete in *B. endophyticus* and plasmids also harbor some sporulation-related proteins, including AbrB family transcription regulatory proteins, spores coat protein and germination proteins GerKA, GerKC and GerKB. *B. endophyticus* has abundant transcriptional regulatory proteins in response to changes of external environment, and the plasmids harbor sigma-W, sigma-B and two-component signal transduction system for the regulation of cell density. In addition, it contains a large number of other transcription factors, covering MerR, ArsR, MarR, AbrB, GntR, AraC and LacI family. Besides, *B. endophyticus* also encodes five cell division proteins and 36 mobile element proteins on plasmids for cell division and proliferation.

### The different morphologies and companion effects of two *Bacillus* species

**Figure 1:**
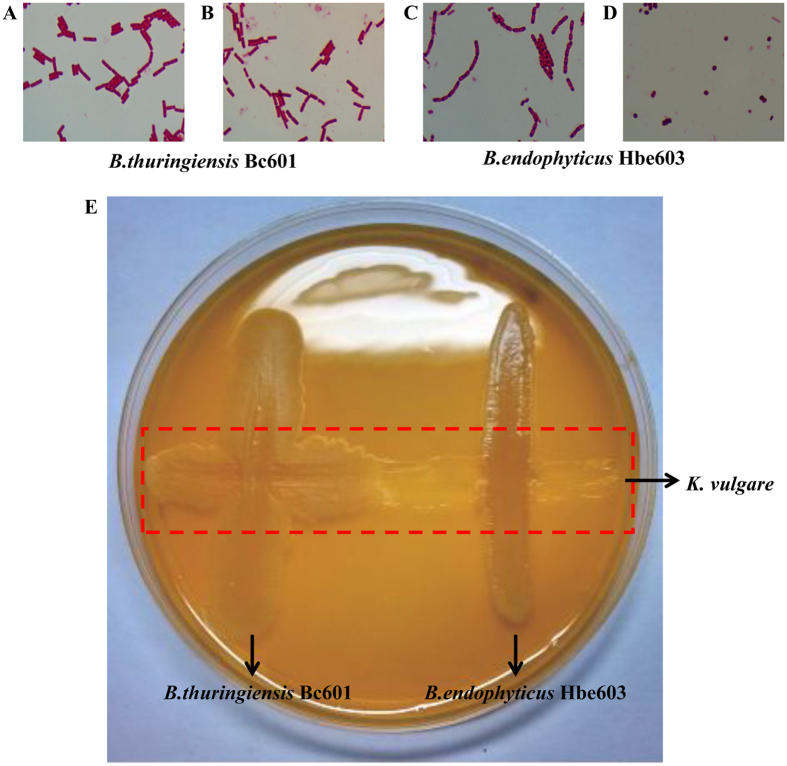
The growth state of *B. endophyticus* Hbe603 and *B. thuringiensis* Bc601 by microscopic observation. The vegetative state (**A**) and sporulation (**B**) of *B. thuringiensis* Bc601 and the vegetative state (**C**) and sporulation (**D**) of *B. endophyticus* Hbe603. Swarming pattern of the ecosystem via chemotaxis of species (**E**). The photographs show monocultures of *B. thuringiensis*, *B. endophyticus* and *K. vulgare* and coculture after 96 hours of growth at 30 °C on soft agar.

**Figure 2:**
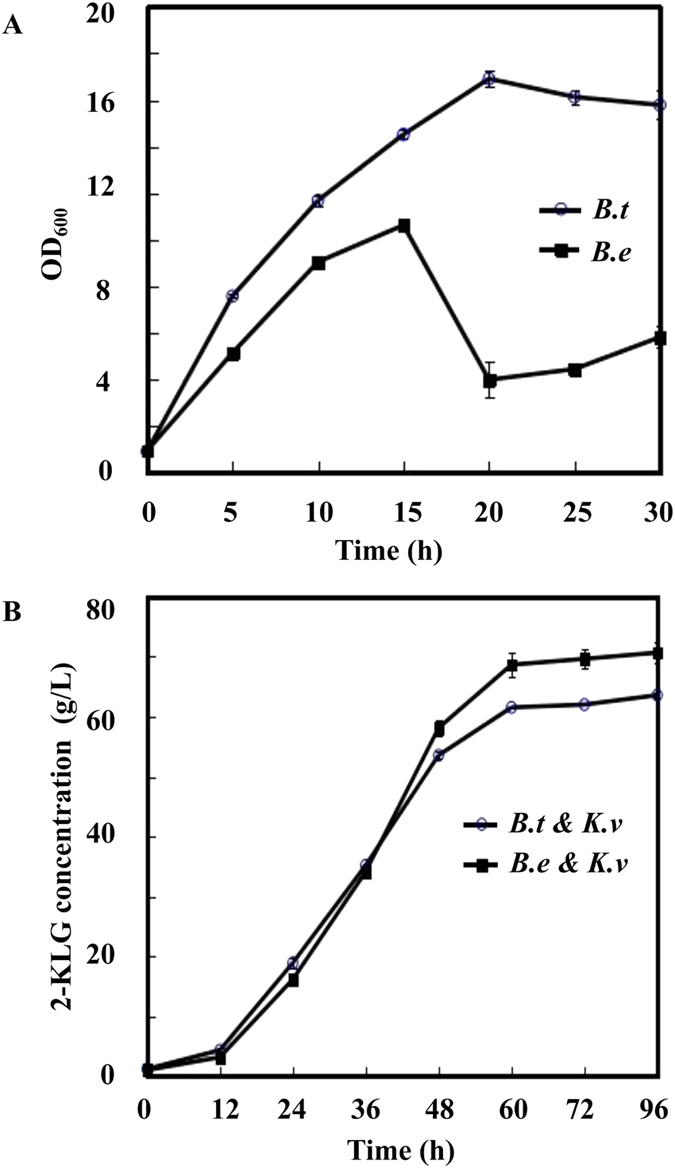
The two *Bacillus* species cooperated with *K. vulgare* in fermentation, respectively. (**A**) Cell density of *Bacillus* species, the Y axis represents the average OD_600nm_ at each time point; (**B**) Concentration of 2-KLG.

By microscopic observation, we found the obvious differences in the morphology of two *Bacillus* species. *B. thuringiensis* has a long rod-shaped with no obvious changes before and after sporulation (Fig. 1A,B). *B. endophyticus* keeps the long chain distribution characteristics in the vegetative state, and becomes elliptic and reduces half of its volume after sporulation (Fig. 1C,D). We speculate that its cell structure is changeable. The different morphologies after sporulation may be connected with the characteristics of two species, particularly the cell structure related proteins. The highest cell density of single-cultured *B. thuringiensis* was 1.59 times of single-cultured *B. endophyticus*, and the cell density decreased significantly after 20 hours due to the sporulation (Fig. 2A). *B. thuringiensis* may absorb more nutrients for its strong growth ability compared to *B. endophyticus* in the mixed bacteria system. In the co-culture system, the 2-KLG productivity of *K. vulgare* with *B. endophyticus* was higher than that with *B. thuringiensis* after 36 hours and tended to be stable in 72 hours (Fig. 2B). Swarming motility is a multi-cellular behavior which can help us to better understand the metabolic interaction and the cooperative mechanism between the two species. We orthogonal cultured *B. thuringiensis*, *B. endophyticus* and *K. vulgare* on an agar plate. The horizontal cultivation was *K. vulgare*, and vertical cultivation was *B. thuringiensis* Bc601 and *B. endophyticus* Hbe603. Swarming motility of *B. thuringiensis* could be induced when it was co-cultured with *K. vulgare*, while *B. endophyticus* didn’t swarm to *K. vulgare* (Fig. 1E).

### Comparative analysis of the versatile metabolism in two *Bacillus* species

To gain further insight into the relationship between *B. thuringiensis* Bc601 and *B. endophyticus* Hbe603, the common and unique genes were calculated using the CD-HIT rapid clustering^20^. *B. thuringiensis* Bc601 and *B. endophyticus* Hbe603 have 1524 common genes, and the number of specific genes is 4189 and 3698, respectively. The distribution of COG classification was compared to facilitate the difference of gene function in two species (Table S3). In *B. thuringiensis* Bc601, the number of genes related to carbohydrate transport and metabolism (G), Energy production and conversion (C) and lipid metabolism (I) is less than that in *B. endophyticus* Hbe603, respectively. We speculate that *B. endophyticus* Hbe603 may have greater promotion for *K. vulgare* due to greater capacity of nutritional supplements. From our observation, the cell wall and membrane structure of *B. thuringiensis* Bc601 are significantly thicker than *B. endophyticus* Hbe603, and the ratio of peptidoglycan biosynthesis related gene number is 29:21. Only *B. thuringiensis* Bc601 harbors *femX*, *femA*, *femB*, *murN*, *mtgA*, *pbpC* and *pbpB*. Besides, D-alanyl-D-alanine carboxypeptidases, collagenases, ATPases, spore coat and two-component system related proteins in *B. thuringiensis* Bc601 are more than those in *B. endophyticus* Hbe603. We speculate these factors may affect *B. endophyticus* Hbe603’s ability to communicate with the external environment. Furthermore, *B. endophyticus* Hbe603 has more PTS related transporters and ferredoxins than *B. thuringiensis* Bc601 (Table 1).

**Figure 3:**
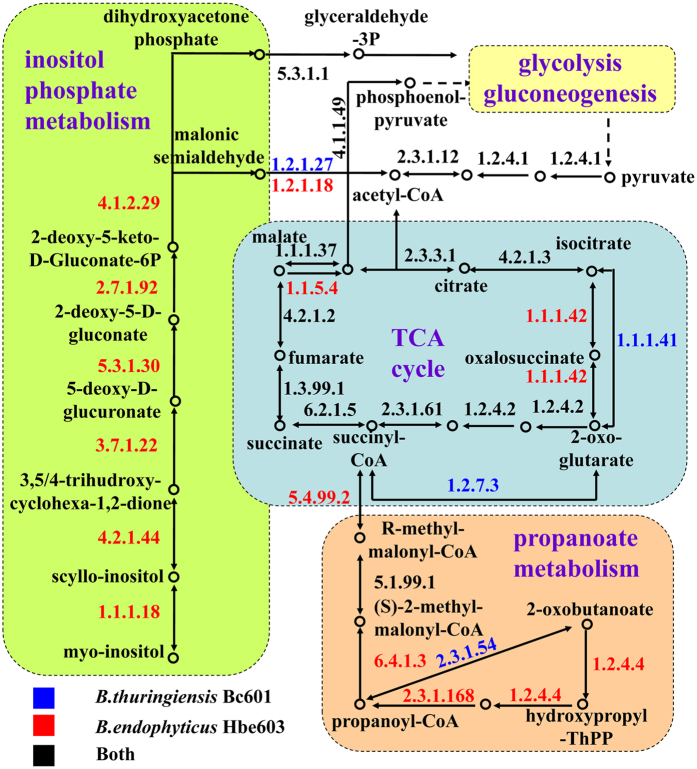
Comparative analysis of the mainly different pathway related to TCA cycle between *B. endophyticus* Hbe603 and *B. thuringiensis* Bc601.

Based on the transcriptome analysis of *B. thuringiensis* sporulation process, 1646 genes were differentially expressed and most of them were connected with transport, transcriptional regulation, cell motility and DNA repair^21^. In the process of sporulation, the companion *Bacillus* bacterium further releases abundant nutrients for the growth and 2-KLG production of *K. vulgare*^22^. In order to analyze the different metabolic capacities in *B. thuringiensis* Bc601 and *B. endophyticus* Hbe603, metabolic network of two species was obtained, including the central carbon, amino acid and cofactor metabolism (Fig. 3).

In the central carbon metabolism, we identified the complete glycolysis, citrate cycle (TCA cycle) and pentose phosphate pathway in the two species. Besides, *B. thuringiensis* Bc601 owns glyceraldehyde-3-phosphate dehydrogenases and 2- oxoglutarate ferredoxin oxidoreductase, which converses glyceraldehyde-3-phosphate to glycerate-3-phosphate, and converts 2-oxoglutarate into succinyl-CoA, respectively. In *B. thuringiensis* Bc601, the alpha-galactosidases, pentose and glucuronate conversion related proteins, which can utilize D-galacturonate, D-altronate, D-mannonate, xylitol, xylose and galactose for carton implying, are less than that in *B. endophyticus* Hbe603. In the propanoate metabolism, only *B. endophyticus* Hbe603 harbors a complete pathway of six steps to converse 2-oxobutanoate to succinyl-CoA, thus implies for the citrate cycle. The absence of propionyl-CoA carboxylase and methylmalonyl-CoA mutase in *B. thuringiensis* Bc601 significantly impedes the metabolic flux to citrate cycle. Particularly, three methylmalonyl-CoA mutases are identified in *B. endophyticus* Hbe603, which have only been found in *B. megaterium*, *Geobacillus kaustophilus* and *Bacillus halodurans*.

In the amino acid metabolism, *B. endophyticus* Hbe603 has more lysine degradation related genes than *B. thuringiensis* Bc601, the ratio of gene number is 26:18. In the tyrosine metabolism, the two species are both lack of adequate genes. *B. thuringiensis* Bc601 uses 4- hydroxyphenylpyruvate dioxygenase and homogentisate 1, 2-dioxygenase to converse tyrosine to 4-maleyl-acetoacetate, and phenylalanine-4-hydroxylase to converse phenylalanine to tyrosine. *B. endophyticus* Hbe603 uses tyrosinase (EC 1.14.18.1) to converse tyrosine to pheomelanin and eunelanin. In the tryptophan metabolism, the two species are both lack of the degradation pathway. They use tryptophan 2, 3- dioxygenase and kynureninase to converse tryptophan to formyl-anthranilate. The two species both have the complete phenylalanine, tyrosine and tryptophan biosynthesis pathway while only *B. thuringiensis* Bc601 has the phenylalanine-4-hydroxylase to converse the phenylalanine to tyrosine. The glutathione metabolism in *B. thuringiensis* Bc601 is not complete and *B. endophyticus* Hbe603 contains five gamma-glutamyl-transpeptidases to converse glutathione to glycine, cysteine and glutamate.

In the cofactor and vitamin metabolism, the two species both have the complete biosynthesis pathway of folate, protoheme, pantothenate and CoA, while the lipoic acid and biotin biosythensis pathway are defect. In the inositol phosphate metabolism, only *B. endophyticus* Hbe603 contains a complete pathway of thirteen key enzymes, including myo-inositol 2-dehydrogenase, inosose dehydratase, glucuronate isomerases, 5-dehydro-2-deoxygluconokinase, 6-phospho-5-dehydro-2-deoxy-D-gluconate aldolase, methylmalonate-semialdehyde dehydrogenase and triosephosphate isomerase. That pathway converses inositol to acetyl-CoA and glyceraldehyde-3-phosphate, which participates in TCA cycle and glycolysis, respectively. In the nicotinate and nicotinamide metabolism, the two species both can transfer L-aspartate to nicotinate, but only *B. thuringiensis* Bc601 contains three nucleotidases that can transfer L-aspartate to nicotinamide.

## Discussion

**Figure 4:**
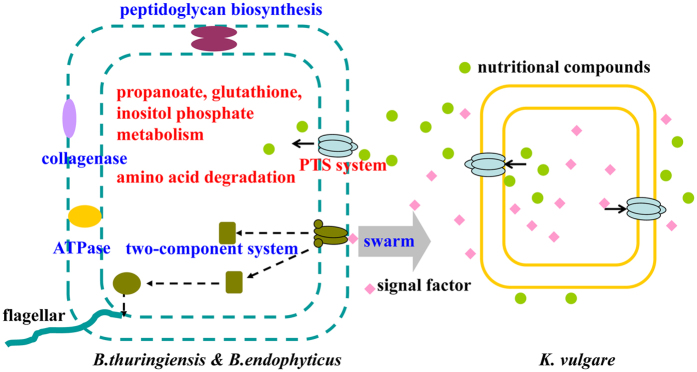
Schematic representation of the companion mechanisms of *Bacillus thuringiensis* Bc601 and *Bacillus endophyticus* Hbe603 in bacterial consortium at a genome-wide scale. For the ability of response to the external environment, *B. thuringiensis* Bc601 has more collagenases, ATPases, two-component system and peptidoglycan biosynthesis related proteins than *B. endophyticus* Hbe603 (marked in blue words). For the ability of nutrients biosynthesis, *B. endophyticus* Hbe603 has more PTSsystem, propanoate, glutathione and inositol phosphate metabolism, and amino acid degradation related proteins than *B. thuringiensis* Bc601 (marked in red words).

Microorganisms can often utilize and secrete a large number of metabolites. This plastic network is readily adapted and regulated in response to nutrients. Swarming motility is a multi-cellular behavior which can help us to better understand the metabolic interaction and cooperative mechanism^23^,^24^. Swarming motility of *B. thuringiensis* could be induced when it was co-cultured with *K. vulgare*, while *B. endophyticus* didn’t swarm to *K. vulgare.* The metabolic interaction and companion mechanism of the two *Bacillus* species are completely different in bacterial consortium (Fig. 4).

By the means of metabolomics, the metabolites changes were identified by *B. thuringiensis*, *K. vlugare* and consortium^10^. The contents of nutritional compounds in the medium surrounding *K. vulgare* were fairly higher than in fresh medium. Erythrose, erythritol, guanine and inositol accumulated around *B. thuringiensis* were consumed by *K. vulgare*, and the oxidization products of *K. vulgare* were sharply increased. For the ability of response to the external environment, *B. thuringiensis* Bc601 has more two-component system, sporulation coat and peptidoglycan biosynthesis related proteins than *B. endophyticus* Hbe603. *B. thuringiensis* is capable of crawling along *K. vulgare*, indicating that it has a stronger ability to communicate to the external environment and respond to the nutrients surrounding *K. vulgare*.

Our previous research showed that the sub-cultivated *B. thuringiensis* and *K. vulgare* significantly increased the productivity of 2-keto-L-gulonic acid^25^. By culturing the *B. thuringiensis* and *K. vulgare* orthogonally on agar plates, the swarming distance of *B. thuringiensis* along the trace of *K. vulgare* decreased after 150 days’ sub-cultivation^26^. Metabolomic analysis showed that the ability of nutrients searching and intaking was increasing in the evolved *B. thuringiensis* and it provided more nutrients to *K. vulgare*. For the ability of nutrients biosynthesis, *B. endophyticus* Hbe603 has more alpha-galactosidases, propanoate, glutathione and inositol phosphate metabolism, and amino acid degradation related proteins than *B. thuringiensis* Bc601. *B. endophyticus* didn’t swarm to *K. vulgare*, probably due to its adequate metabolic capacity in consortium. The high production of 2-KLG is also connected with the abundant nutrition that *B. endophyticus* provided.

Although thousands of *B. thuringiensis* strains were isolated, less than 30 complete genomes were obtained. The complete genome sequencing of *B. thuringiensis Bc601* adds a new member in genome library of *B. thuringiensis* family, and the genomic analysis will give us the opportunity to investigate the diversity and evolution among *B. thuringiensis*-*B. cereus* family. *B. endophyticus* Hbe603 is the first in that species with complete genome, which will provide the important genetic background and molecular information for gene modification. All in all, comparative genomic analysis of *B. endophyticus* and *B. thuringiensis* enables us to identify the unique genes for each species and understand the companion mechanism for system optimization.

## Methods

### Bacteria and cultivation conditions

The growth medium was composed of 2% L-sorbose, 0.3% corn-steep liquor (CSL), 1% peptone, 0.3% yeast extract, 0.3% beef extract, 0.1% urea, 0.1% KH_2_PO_4_, 0.02% MgSO_4_·7H_2_O and 0.1% CaCO_3_. The fermentation medium contained 8% L-sorbose, 2% corn-steep liquor (CSL), 1.2% urea, 0.1% KH_2_PO_4_, 0.05% MgSO_4_.7H_2_O and 0.1% CaCO_3_. The seed cultures of *K. vulgare* and *Bacillus* species were cultivated in 250 mL flasks with 50 mL growth medium (30 °C, 250 rpm) for 24 hours. Subsequently, the two species were co-inoculated into 250 mL flasks with 50 mL fermentation medium at 250 rpm, 30 °C for 96 hours. Swarming motility was observed by culturing the two *Bacillus* species with *K. vulgare* orthogonally on agar plates. *Bacillus* species and *K. vulgare* were cultivated for 12 hours and 36 hours, respectively, and then inoculated separately in solid sorbose-CSL medium containing 1.5% agar and cultivated at 30 °C for 96 hours.

### Analyses of 2-KLG and biomass

The concentration of extracellular 2-KLG were determined by the High Performance Liquid Chromatography (HPLC) (Waters Corp., Massachusetts, USA), equipped with an Aminex HPX-87H column (Bio-Rad, CA) and a refractive index detector. The mobile phase used in the HPLC system was 5 mM H_2_SO_4_ at 65 °C with a flow rate of 0.6 mL/min. The cell density was measured as optical density at 600 nm (OD_600_) with a spectrophotometer after dissolving CaCO_3_ in 100 mM HCl.

### DNA extraction, genome sequencing and assembly

Isolation of genomic DNA was carried out using SDS method. Total DNA obtained was subjected to quality control by agarose gel electrophoresis and quantified by Qubit. DNA was used to construct a 10 kb SMRTbell library, and the genome was sequenced by Single Molecule, Real-Time (SMRT) technology. Sequencing was performed at the Beijing Novogene Bioinformatics Technology Co., Ltd. SMRT Analysis 2.3.0 were used to filter low quality reads and the filtered reads were assembled by SOAPdenovo (http://soap.genomics.org.cn/soapdenovo.html) to generate the complete genome, which has been confirmed by PCR amplification.

### Gene prediction, annotation and protein classification

Gene prediction was performed on the genome assembly by GeneMarkS^27^. Transfer RNAs (tRNAs) were predicted with tRNAscan-SE^28^, ribosome RNAs (rRNAs) were predicted with rRNAmmer^29^ and sRNAs were predicted by BLAST against Rfam database^30^. PHAST^31^ was used for prophage prediction and CRISPRFinder^32^ was used for CRISPR identification. A whole genome Blast^33^ search was performed against KEGG database (Kyoto Encyclopedia of Genes and Genomes)^34^, COG database (Clusters of Orthologous Groups)^35^, NR database (Non-Redundant Protein Database) and Swiss-Prot database^36^. The origin of replication (*oriC*) and putative DnaA boxes were identified by Ori-Finder^37^. CVTree was performed for the phylogenetic analysis^38^ and the phylogenetic tree was generated using the MEGA program^39^. The GC-Profile was used to compute the GC content variation in DNA sequences and to predict the horizontal gene transfer^40^. CGView Server was used for the visualization of circular genomes^41^ and the metabolic network was constructed by KEGG automatic annotation server KAAS^42^.

### Nucleotide sequence accession numbers

The genome sequence of the *B. thuringiensis* Bc601 has been deposited at DDBJ/EMBL/GenBank under the accession numbers CP015150 (chromosome), CP015151 to CP015156 (six plasmids, respectively). The genome sequence of the *B. endophyticus* HBe603 has been deposited at DDBJ/EMBL/GenBank under the accession numbers CP011974 (chromosome), CP015323 to CP015330 (eight plasmids, respectively).

## Additional Information

**How to cite this article**: Jia, N. *et al.* Comparative genomics analysis of the companion mechanisms of *Bacillus thuringiensis* Bc601 and *Bacillus endophyticus* Hbe603 in bacterial consortium. *Sci. Rep.* **6**, 28794; doi: 10.1038/srep28794 (2016).

## Supplementary Material

Supplementary Information

## Figures and Tables

**Table 1:** The major different genes among two *Bacillus* species.

Gene product	Gene number	
	*B.endophyticus* Hbe603	*B. thuringiensis* Bc601
D-alanyl-D-alanine carboxypeptidase	4	7
collagenase	0	4
spore coat protein	12	7
polysaccharide deacetylase	2	5
PTS system	21	15
two component system	40	50
transport system permease	18	21
aldehyde dehydrogenase	23	8
ferredoxin	7	4
oxidoreductase	48	45
quinol/ubiquinol oxidase	16	9
methylmalonyl-CoA mutase	3	0
ATPase	6	13
