# Supplementary material for: Comparative genomics analysis of the companion mechanisms of Bacillus thuringiensis Bc601 and Bacillus endophyticus Hbe603 in bacterial consortium
Source: Sci Rep. 2016 Jun 29;6:28794. doi: 10.1038/srep28794 (PMC4926094; doi:10.1038/srep28794)
Supplement: Supplementary Information [file srep28794-s1.pdf]

Supplementary Information

Comparative genomics analysis of the companion mechanisms of *Bacillus thuringiensis* Bc601 and *Bacillus endophyticus* Hbe603 in bacterial consortium

Nan Jia , Ming-Zhu Ding *, Feng Gao*, Ying-Jin Yuan

* Corresponding authors: Ming-Zhu Ding, Feng Gao

Email: mzding@tju.edu.cn, fgao@tju.edu.cn

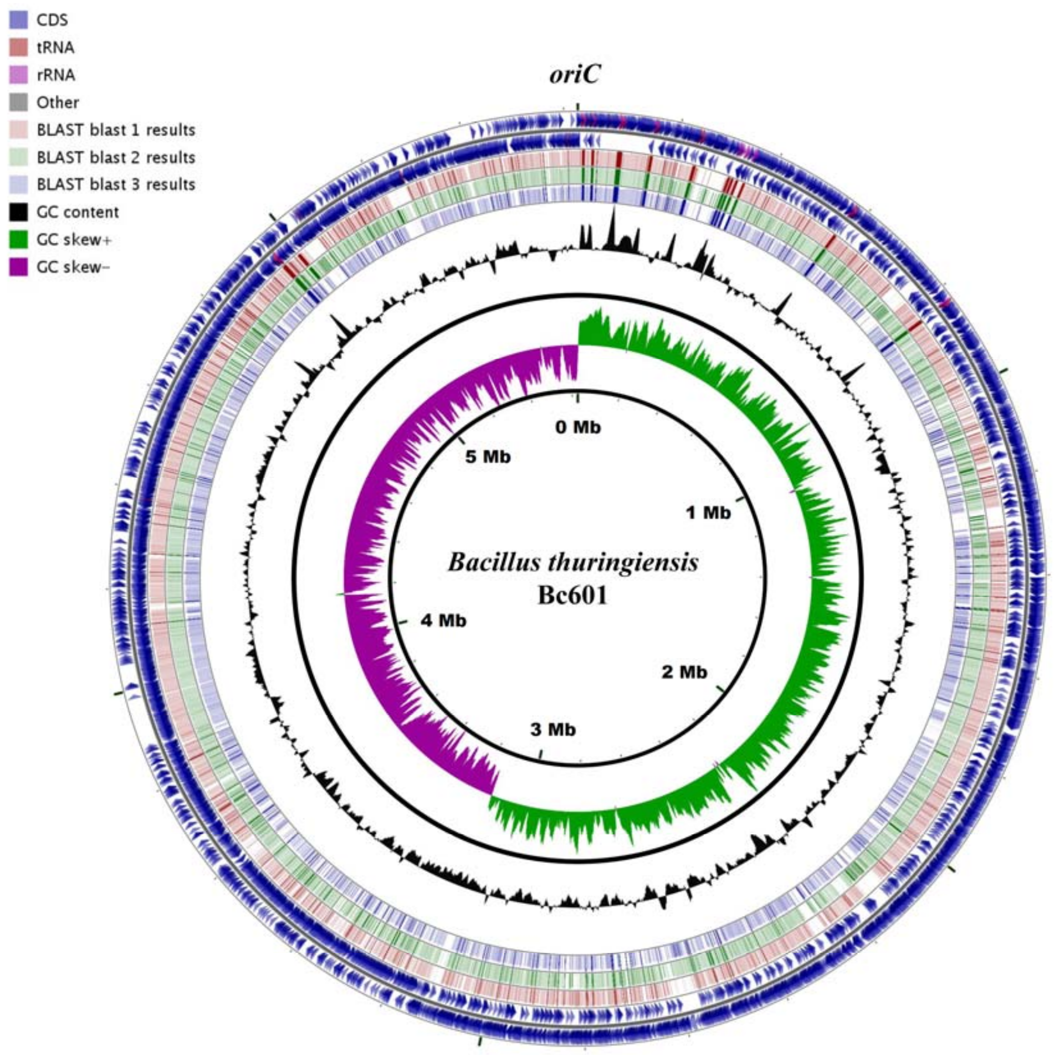

Figure S1 The genome schematic of *B. thuringiensis.* Circles from the outside to the inside show the positions of protein-coding genes (blue), tRNA genes (red) and rRNA genes (pink) on the positive (circle 1), and negative (circle 2) strands. Circles 3-5 show the positions of BLAST hits detected through blastx comparisons of *B. thuringiensis* Bc601 against *B. thuringiensis* serovar konkukian str. 97-27 (circle 3), *B. thuringiensis* YBT-1518 (circle 4) and *B. thuringiensis* str. Al Hakam (circle 5). Circles 6 and 7 show plots of GC content and GC skew plotted as the deviation from the average for the entire sequence.

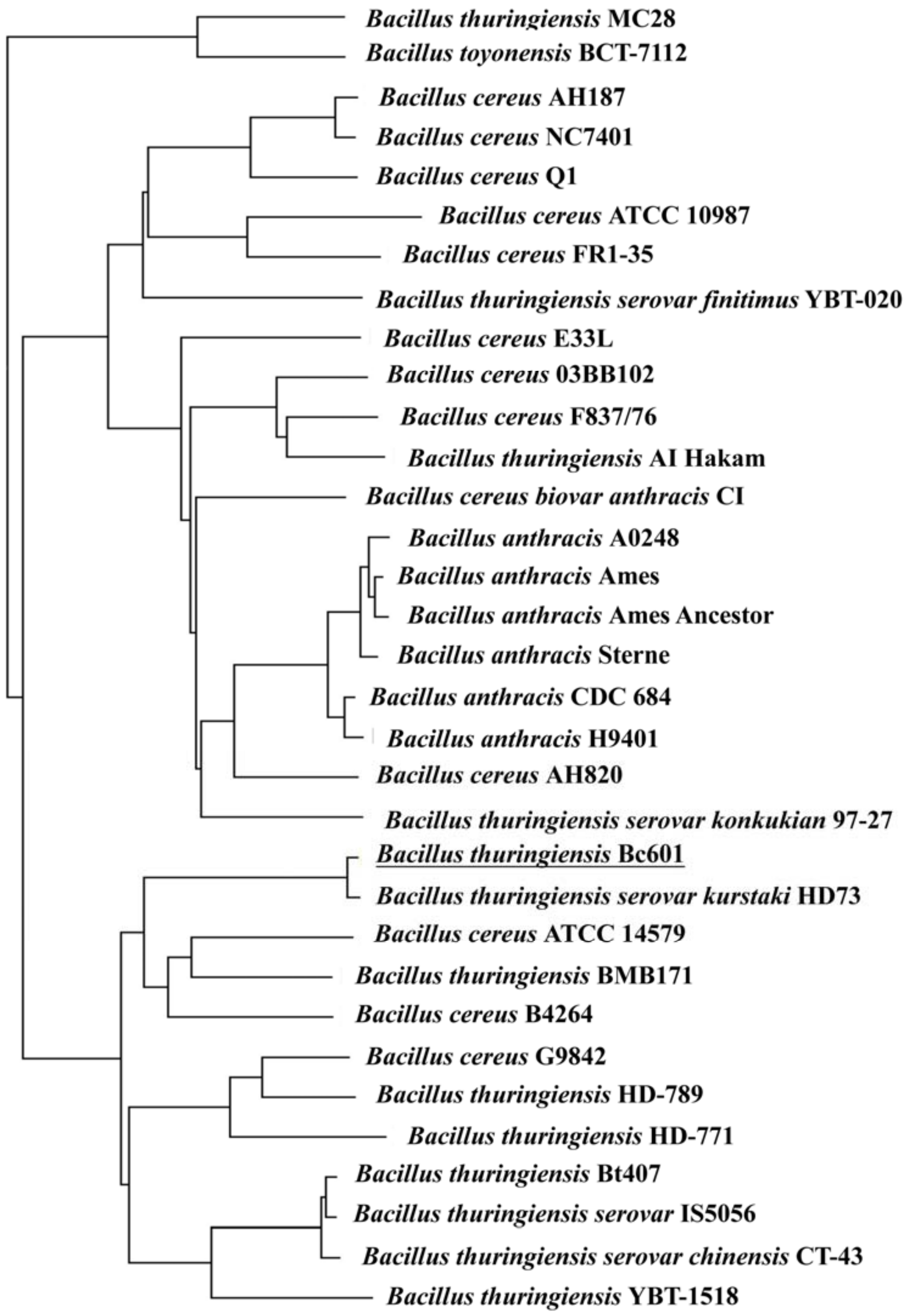

Figure S2 Phylogenetic analysis of *B. thuringiensis* Bc601 with other species. The phylogenetic tree of *B. thuringiensis* Bc601 was constructed using CVTree with parameters K=6 and Type=aa. The neighbor-joining tree was constructed using the MEGA5 program.

Table S1. The statistics of final assembly result in *B. thuringiensis* Bc601.

Name	INSDC	Size (Mb)	GC%	Protein	rRNA	tRNA	Other RNA	Gene
BtBc601	CP015150.1	5.63	35.3	5,485	39	107	5	5,735
pBTBC1	CP015151.1	0.014888	31.1	17	-	-	-	18
pBTBC2	CP015152.1	0.171171	33.7	149	-	-	-	156
pBTBC3	CP015153.1	0.042357	30.9	28	-	-	-	43
pBTBC4	CP015154.1	0.082417	33.6	76	-	-	-	82
pBTBC5	CP015155.1	0.083987	31.2	88	-	-	-	90
pBTBC6	CP015156.1	0.08935	32.5	88	-	-	-	93

Table S2. The statistics of final assembly result in *B.endophyticus* Hbe603.

Name	Accession	Size (Mb)	GC%	Protein	rRNA	tRNA	Other RNA	Gene
BEH	CP011974.1	4.87	36.6	4,634	33	81	5	4,818
pBEH1	CP015323.1	0.095077	35.2	83	-	-	-	92
pBEH2	CP015324.1	0.058079	34.6	48	-	-	-	54
pBEH3	CP015325.1	0.084656	35.5	87	-	-	-	93
pBEH4	CP015326.1	0.06917	35.2	58	-	-	-	59
pBEH5	CP015327.1	0.05675	35.1	47	-	-	-	55
pBEH6	CP015328.1	0.069165	33.3	74	-	-	-	75
pBEH7	CP015329.1	0.010461	35.5	12	-	-	-	12
pBEH8	CP015330.1	0.005053	33.8	5	-	-	-	5

Table S3. Comparison of the COG classification between *B. thuringiensis* and *B.endophyticus*.

COG classification	*B. thuringiensis* Bc601	*B. endophyticus* Hbe603
C, Energy production and conversion	179	248
D, Cell division and chromosome partitioning	31	64
E, Amino acid transport and metabolism	341	395
F, Nucleotide transport and metabolism	93	105
G, Carbohydrate transport and metabolism	197	356
H, Coenzyme metabolism	132	232
I, Lipid metabolism	104	176
J, Translation, ribosomal structure and biogenesis	183	234
K, Transcription	294	388
L, DNA replication, recombination, and repair	187	126
M, Cell envelope biogenesis, outer membrane	147	192
N, Cell motility and secretion	37	44
O, Posttranslational modification, protein turnover, chaperones	94	124
P, Inorganic ion transport and metabolism	216	218
Q, Secondary metabolite biosynthesis, transport, and catabolism	81	135
R, General function prediction only	436	436
S, Function unknown	285	233
T, Signal transduction mechanisms	135	194
U, Intracellular trafficking and secretion	44	34
V, Defense mechanisms	76	111
